# A Combined Barite–Ilmenite Weighting Material to Prevent Barite Sag in Water-Based Drilling Fluid

**DOI:** 10.3390/ma12121945

**Published:** 2019-06-17

**Authors:** Salem Basfar, Abdelmjeed Mohamed, Salaheldin Elkatatny, Abdulaziz Al-Majed

**Affiliations:** College of Petroleum Engineering and Geosciences, King Fahd University of Petroleum & Minerals, 31261 Dhahran, Saudi Arabia; g201407960@kfupm.edu.sa (S.B.); abdelmjeed.mohamed@kfupm.edu.sa (A.M.); aamajed@kfupm.edu.sa (A.A.-M.)

**Keywords:** barite, ilmenite, combined weighting agent, barite sag, water-based drilling fluids, HPHT wells

## Abstract

Barite sag is a serious problem encountered while drilling high-pressure/high-temperature (HPHT) wells. It occurs when barite particles separate from the base fluid leading to variations in drilling fluid density that may cause a serious well control issue. However, it occurs in vertical and inclined wells under both static and dynamic conditions. This study introduces a combined barite–ilmenite weighting material to prevent the barite sag problem in water-based drilling fluid. Different drilling fluid samples were prepared by adding different percentages of ilmenite (25, 50, and 75 wt.% from the total weight of the weighting agent) to the base drilling fluid (barite-weighted). Sag tendency of the drilling fluid samples was evaluated under static and dynamic conditions to determine the optimum concentration of ilmenite which was required to prevent the sag issue. A static sag test was conducted under both vertical and inclined conditions. The effect of adding ilmenite to the drilling fluid was evaluated by measuring fluid density and pH at room temperature, and rheological properties at 120 °F and 250 °F. Moreover, a filtration test was performed at 250 °F to study the impact of adding ilmenite on the drilling fluid filtration performance and sealing properties of the formed filter cake. The results of this study showed that adding ilmenite to barite-weighted drilling fluid increased fluid density and slightly reduced the pH within the acceptable pH range (9–11). Ilmenite maintained the rheology of the drilling fluid with a minimal drop in rheological properties due to the HPHT conditions, while a significant drop was observed for the base fluid (without ilmenite). Adding ilmenite to the base drilling fluid significantly reduced sag factor and 50 wt.% ilmenite was adequate to prevent solids sag in both dynamic and static conditions with sag factors of 0.33 and 0.51, respectively. Moreover, HPHT filtration results showed that adding ilmenite had no impact on filtration performance of the drilling fluid. The findings of this study show that the combined barite–ilmenite weighting material can be a good solution to prevent solids sag issues in water-based fluids; thus, drilling HPHT wells with such fluids would be safe and effective.

## 1. Introduction

Drilling fluid contains a mixture of solids and polymers. Solids such as weighting materials are used mainly to increase the mud density, while clays and polymers are used to control the rheological and filtration properties. For bentonite-based drilling fluid, the rheological and filtration properties can be improved by using chitin nanocrystals from speckled swimming crab shell waste at different ranges of pH [1] or by adding cellulose nanocrystals, especially in the case of low solid content of bentonite–water-based drilling fluid [2]. In addition, Wyoming sodium bentonite–water-based drilling fluid has better suspension and filtration control properties when mixing nanoparticles with bentonite particles in different configurations [3].

Polymers are used heavily in the oil industry for controlling the drilling fluid properties or for enhanced oil recovery applications to extract the heavy oil. Polymer flooding is a very important technique to extract the heavy oil from thin and heterogeneous reservoirs [4]. Screening criteria and screening algorithms should be developed for enhanced oil recovery techniques [5]. Artificial intelligence and data mining can be used to manage the reservoir for polymer flooding, especially in case of thin and heterogeneous heavy oil reservoirs [6].

Drilling high-pressure/high-temperature (HPHT) oil and gas wells is challenging because it requires a special fluid formulation that can control the high pressure and withstand the elevated downhole temperatures [7]. Weighting materials are added to the drilling fluid to attain the high density required to equalize formation pressure and control the well during drilling operations [8,9,10,11]. There are many options for weighting materials that can be used with drilling fluids such as calcite, barite, hematite, manganese tetra oxide, ilmenite, and iron oxide [12,13,14,15,16,17,18]. These materials are different in density and other properties; therefore, the final density of drilling fluid varies accordingly [10,19]. 

**Figure 1:**
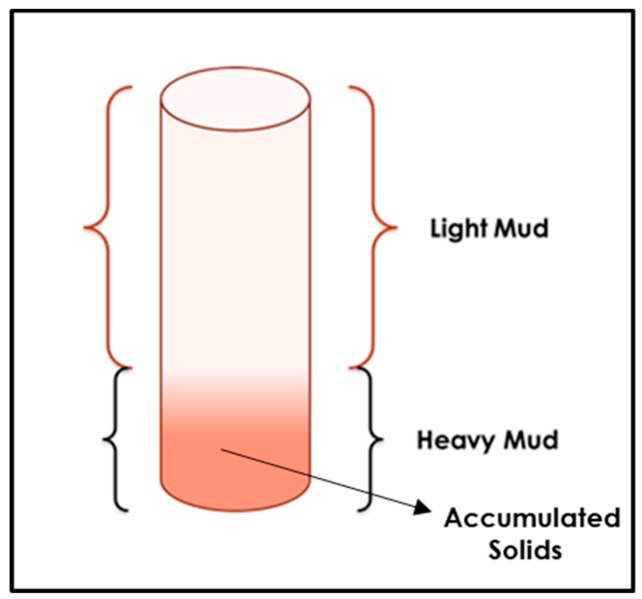
Solids sag phenomenon.

Barite is a common weighting material used to formulate high-density drilling fluids because of its high density (4.2–4.48 g/cm^3^), cheap cost, and low environmental impact. Suspension of weighting agents is critical because settled particulates will not contribute to the hydrostatic column of the drilling fluid, leading to serious well control issues (Figure 1). This phenomenon is known as solids sag or barite sag because it is mostly encountered when drilling with barite-weighted fluids [9,20]. However, barite sag can occur in vertical and directional wells and under dynamic and static conditions, and it is worse in dynamic conditions [21]. Barite sag can be mitigated by optimizing drilling fluid rheology, implementing sound strategies, and training rig personnel [22].

Many research studies were conducted to understand and prevent the sag phenomenon in oil- and water-based drilling and completion fluids. A new method was proposed by Temple et al. [23] to prevent barite sag in oil-based drilling fluid by adding polyalkyl methacrylate, which has a low molecular weight, while no copolymer was added; therefore, no additional increase in drilling fluid viscosity would occur. Alabdullatif et al. [24] introduced a new water-based kill fluid to avoid the barite sag problem. Barite–Mn_3_O_4_ was used as combined weighting agent to increase the density of the kill fluid. They concluded that the addition of Mn_3_O_4_ effectively enhanced fluid stability and reduced barite sag, particularly after a long time under static conditions. 

Mohamed et al. [7] investigated the effect of barite particle size on water-based drilling fluid stability using a wide range of particle size. They found that reducing barite particle size to a micronized size (around 8 μm) slightly enhanced the stability of the drilling fluid; however, it did not prevent barite sag. Davis et al. [25] proposed a new fluid formulation consisting of base fluid (invert emulsion), weighting material (barite), and sag stability enhancer. The sag stability enhancer comprised polyethylene glycol (PEG) having a molecular weight greater than or equal to 200 g/mol [25]. 

Basfar et al. [26] and Elkatatny [27] studied the effect of adding a new copolymer on sag tendency of invert emulsion drilling fluid at different temperatures. They concluded that adding only 1 lbm/bbl of copolymer to the drilling fluid prevents barite sag in both vertical and inclined conditions at a temperature up to 350 °F. Elkatatny [28] evaluated organophilic phyllosilicate (OP) as a solution to barite sagging in invert emulsion drilling fluid at elevated temperature (350 °F). He found that adding only 1.5 g of organophilic phyllosilicate was adequate to prevent barite sagging under dynamic conditions.

Because of the high demand and limited resources of barite, many research studies were conducted to find another dense weighting material that can be used to drill HPHT oil and gas wells [29]. Ilmenite is one of the weighting materials that can be used for that purpose because of its high density (4.7–4.79 g/cm^3^), good performance, availability, and acidity [13,30]. Ilmenite was introduced to the oil industry as a weighting agent in 1976; however, it was not commonly used because it is abrasive to drilling equipment [17]. To avoid the abrasiveness of ilmenite, the particle size distribution should be optimized, and less than 3% of the large particles (>45 μm) should be used [29].

Elkatatny et al. [31] evaluated micronized ilmenite as a weighting agent to be used in water-based drilling fluids. They concluded that ilmenite-weighted fluids can be a good option for HPHT applications and no solids sag was observed. Xiao et al. [17] conducted another study using micronized ilmenite with oil-based drilling fluids. They found that ilmenite can be used successfully in oil-based fluids with reduced sag tendency. Another study was also conducted by mixing ilmenite with barite to be used as weighting material. The effect of using this mixture on drilling fluid density and rheology was studied by Abdou et al. [32]. They concluded that this mixture can replace barite because it yields comparable results with lower solids content, which would reduce the formation damage. 

This study introduces a combined (barite–ilmenite) weighting material as a new solution to the barite sag issue in water-based drilling fluids to be used for drilling HPHT oil and gas wells. The effect of using the combined weighting material on all drilling fluid properties was evaluated in this study. Using the combined weighting agent would reduce sag tendency of barite-weighted fluids and minimize the cost of ilmenite-weighted fluids. Firstly, the methodology followed to conduct this study is presented. Then, the results of this study are discussed. Finally, the findings of this research work are concluded. 

## 2. Methodology

### 2.1. Materials

**Figure 2:**
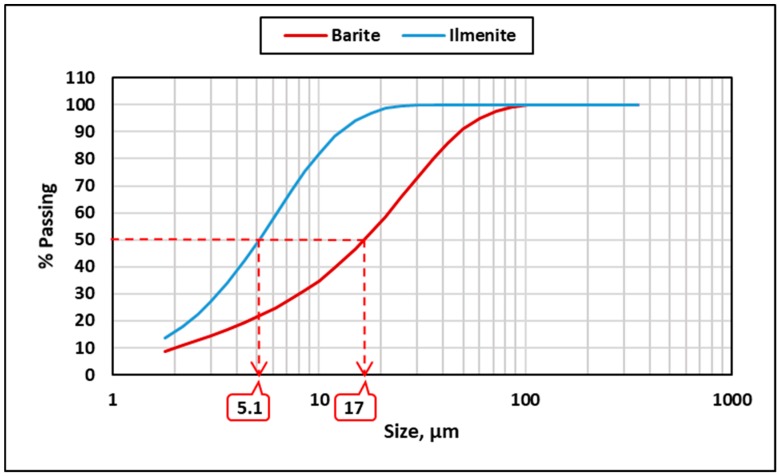
Particle size distribution for the used weighting materials.

Water-based drilling fluid was formulated by mixing the main drilling fluid additives, obtained from local suppliers, at room temperature using a three-speed mixer. These additives were viscosity and pH control additives, a clay stabilizer, a bridging agent, and the weighting material. Barite was used as a weighting agent to attain the high density (15 ppg) of the base drilling fluid. Then, different fluid samples were prepared using a combination of barite and ilmenite with different percentages of ilmenite (25, 50, 75, and 100%) from the total weight of the weighting material. Table 1 describes the drilling fluid formula used in this study, and Table 2 shows the properties of the base drilling fluid (100 wt.% barite). The barite used in this study had an average particle size (D_50_) of around 17 μm, while ilmenite had an average particle size of around 5 μm. Figure 2 compares the particle size distribution of both barite and ilmenite. 

### 2.2. Static Sag Test

**Figure 3:**
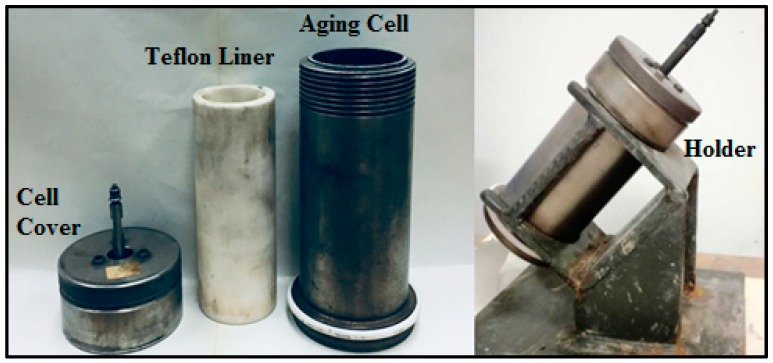
Static sag test set-up [28].

A static sag test was performed to study the effect of ilmenite on sag tendency of barite-weighted drilling fluid in static conditions. The test was conducted in vertical and inclined conditions (45°) because the solids sag issue becomes more serious at an inclination angle above 30° [33]. Figure 3 shows the experimental set-up used to perform the static sag test. It consisted of a Teflon liner, aging cell, and a holder to keep the cell at 45° during the test. The test was performed at 250 °F and 500 psi. Pressure was applied using nitrogen gas. The experimental conditions for the static sag test are shown in Table 3. 

After 24 h, two fluid samples (10 mL) were collected form the top and the bottom of the fluid and weighted; then, the sag factor was calculated using Equation (1). The acceptable sag factor ranges from 0.50 to 0.53 [24,34].

(1) $$\mathrm{Sag}\;\mathrm{Factor}=\frac{w_{Bottom}}{w_{Bottom}+w_{Top}}.$$

### 2.3. Dynamic Sag Test

**Figure 4:**
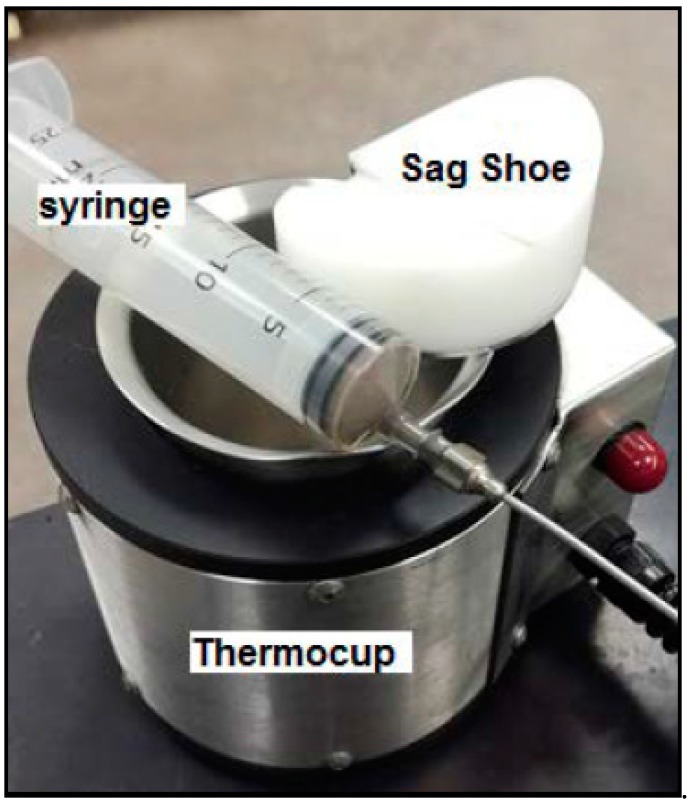
Experimental set-up for viscometer sag shoe test (VSST) excluding the viscometer [27].

The sag tendency of drilling fluid samples was evaluated under dynamic conditions using a viscometer sag shoe test (VSST). The test was conducted in standard conditions, i.e., atmospheric pressure and 120 °F (Table 2). Figure 4 shows the experimental set-up used to conduct the dynamic sag test. The procedure followed to conduct the test was as follows:

- The sag shoe was put in the bottom of the cup and the drilling fluid sample (140 mL) was poured in the cup;
- The fluid sample was heated until 120 °F and a viscometer was run at 100 RPM;
- Then, a 10-mL sample was collected from the collection well in the bottom of the sag shoe using a syringe with a cannula, and the weight was recorded (W_1_);
- After 30 min, the viscometer was stopped, and another sample was taken from the bottom and weighted (W_2_);
- The dynamic sag factor (VSST) was calculated in ppg using Equation (2).

(2) $$VSST=0.834\;\left(W_{2}-W_{1}\right).$$

A drilling fluid that has a VSST value equal to or less than 1.0 ppg will exhibit good performance, and solids sag is unlikely to occur [35], while a VSST value more than 1.6 ppg reflects a bad sag performance, and solids sag is highly anticipated [36].

### 2.4. Measurements of Rheology and Viscoelastic Properties 

Drilling fluid rheology was measured in low- and high-temperature conditions (120 °F and 250 °F) to investigate the effect of adding ilmenite on the rheological properties of the drilling fluid formulation. These rheological properties were yield point, plastic viscosity, and gel strength after 10 s, 10 min, and 30 min. Plastic viscosity (PV) and yield point (YP) were calculated from dial reading data (Ø) at 600 and 300 RPM using Equations (3) and (4), respectively. On the other hand, gel strength data were obtained directly from dial readings at 3 RPM after 10 s, 10 min, and 30 min of gel time.

(3) $$\mathrm{PV}\;\left(\mathrm{cP}\right)=\emptyset_{600}-\emptyset_{300};$$

(4) $$\mathrm{YP}\;\left(\frac{\mathrm{lb}}{100ft^{2}}\right)=\emptyset_{300}-\mathrm{PV}.$$

Viscoelastic properties of drilling fluid samples were measured by conducting an oscillatory amplitude test and an oscillatory frequency test using an Anton Paar 302 rheometer. Firstly, the oscillatory amplitude test was conducted to identify the linear viscoelastic range. The test was run at 250 ℉ with a constant frequency of 0.2 rad/s. Then, the oscillatory frequency test was run to obtain the storage modulus G′ and loss modulus G″. The storage modulus can be an indicative tool to detect solids sag tendency [34].

### 2.5. HPHT Filtration Test

Filtration tests were performed to evaluate the effect of adding ilmenite on the filtration performance of the drilling fluid and the sealing properties of the formed filter cake. The test was performed at 250 °F and 300 psi differential pressure, which was applied using nitrogen gas. A 10-μm ceramic filter disc was used as the filtration medium. The test was started, and the filtrate volume was recorded with time for 30 min. Then, the weight and the thickness of the formed filter cake were measured. Table 4 summarizes the experimental conditions for the HPHT filtration test.

## 3. Results and Discussion

**Figure 5:**
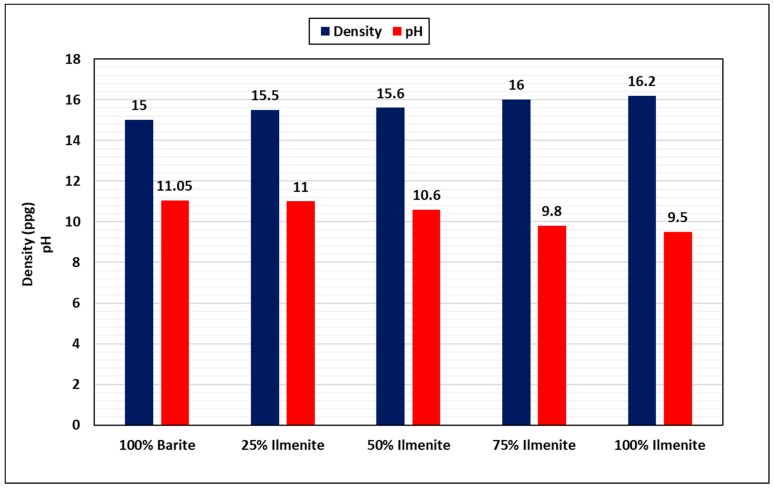
Effect of ilmenite on drilling fluid density and pH at room temperature.

Ilmenite was added to the base barite-weighted drilling fluid with different percentages from the total weighting material (25, 50, and 75 wt.%). Figure 5 shows the effect of adding ilmenite on the density and the pH of the drilling fluid measured at room temperature. The base drilling fluid had a density of 15 ppg and pH of 11. As the concentration of ilmenite increased, the final density of the drilling fluid increased to 16.2 ppg when using 100 wt.% ilmenite. This increase was because of the difference between barite density, 4.48 g/cm^3^, and ilmenite density, 4.79 g/cm^3^ [19]. Therefore, less weighting material is required to formulate a drilling fluid with the same density. In contrast, the pH slightly decreased with the increase of ilmenite concentration, and the pH dropped from 11 for the base fluid to 9.5 for 100% ilmenite. However, this pH is still within the acceptable range for drilling fluids (pH: 9–11) and it can be adjusted to the required value by adding pH control additives such as sodium hydroxide and potassium hydroxide [19].

**Figure 6:**
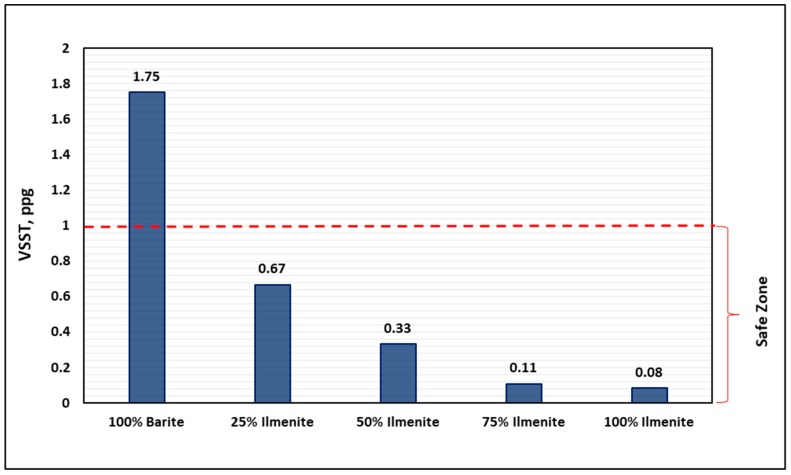
Effect of ilmenite on drilling fluid sag tendency under dynamic conditions (120 °F).

**Figure 7:**
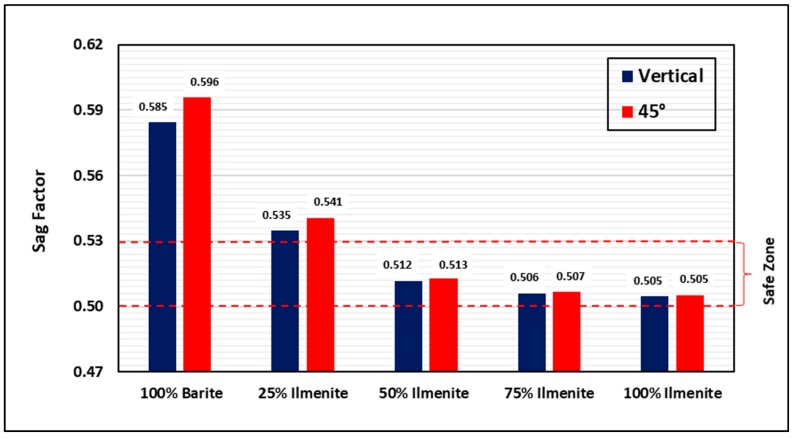
Effect of ilmenite on sag tendency under static conditions (250 °F).

Figure 6 and Figure 7 compare the sag factor of the base fluid (100 wt.% barite) with the other drilling fluid samples (25, 50, 75, and 100 wt.% ilmenite) in dynamic and static conditions, respectively. Under static conditions, the base fluid exhibited bad performance with sag factors of 0.585 and 0.596 in vertical and inclined conditions, respectively, which are above the safe range of 0.50–0.53 [24,34]. Furthermore, the sag factor in dynamic conditions (VSST) was 1.75, which is higher than the acceptable value of 1.6 [36]. Therefore, barite sag is highly anticipated under dynamic and static conditions, which makes drilling with this fluid unsafe. Conversely, it was observed that, for both dynamic and static conditions, as the concentration of ilmenite increased, the sag factor decreased, which indicates an improvement in the performance of the drilling fluid. For dynamic conditions, only 25 wt.% ilmenite was enough to prevent barite sag with a VSST value of 0.67, while, for static conditions, adding 50 wt.% ilmenite prevented barite sag in both vertical and inclined conditions with a sag factor of 0.51.

**Figure 8:**
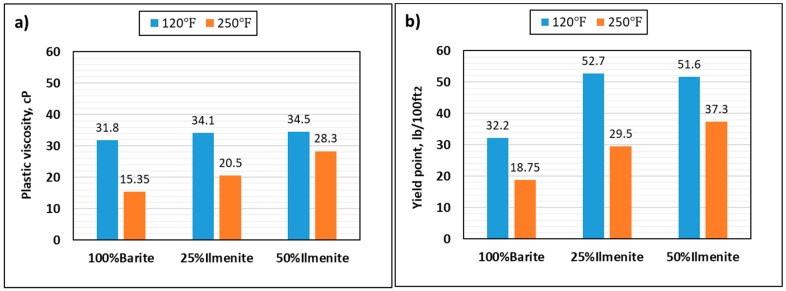
Effect of ilmenite on (**a**) plastic viscosity, and (**b**) yield point.

**Figure 9:**
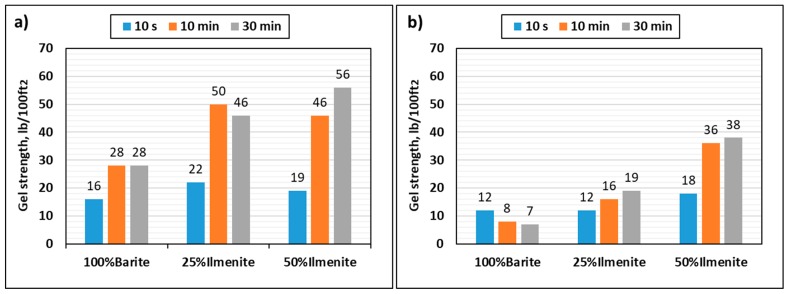
Effect of ilmenite on gel strength at (**a**) 120 °F, and (**b**) 250 °F.

Figure 8 compares the plastic viscosity and yield point of the base fluid, 25 wt.% ilmenite, and 50 wt.% ilmenite at 120 and 250 °F. At 120 °F, adding ilmenite to the drilling fluid increased the yield point up to 52 lb/100 ft2 for 50 wt.% ilmenite, while it was 32 lb/100ft2 for the base fluid. In contrast, the plastic viscosity did not change significantly (from 32 to 34 cP); thus, no more frictional pressure drop would occur during circulation. Many drilling fluid issues are affected by the ratio of yield point to plastic viscosity, such as hole cleaning, barite sag, equivalent circulating density, and surge and swab pressures [37]. Chilingarian et al. [38] proposed a YP/PV ratio to evaluate drilling fluid stability. Higher YP/PV ratios indicate more stabilization. The increase in yield point values, which resulted from adding ilmenite to the drilling fluid, increased the YP/PV ratio, leading to an enhancement of the drilling fluid stability [38]. At 250 °F, huge drops in the plastic viscosity and yield point of the base fluid were observed (51% and 42%, respectively) because of the high temperature, while adding 50 wt.% ilmenite maintained the rheological properties of the drilling fluid with drops of 18% and 27% in PV and YP values, respectively. Gel strength data were obtained from the direct readings at 3 RPM after 10 s, 10 min, and 30 min. These data were measured at 120 and 250 °F. As shown in Figure 9, at high temperature, the base fluid showed a bad performance as gel strength decreased with time; therefore, the base fluid formed an unstable gel that broke with time at elevated temperatures. Conversely, adding ilmenite enhanced the gel strength, which showed a better performance at high temperature. This rheological behavior confirms the sag test results, whereby the 50 wt.% ilmenite fluid sample is more stable than the base fluid.

**Figure 10:**
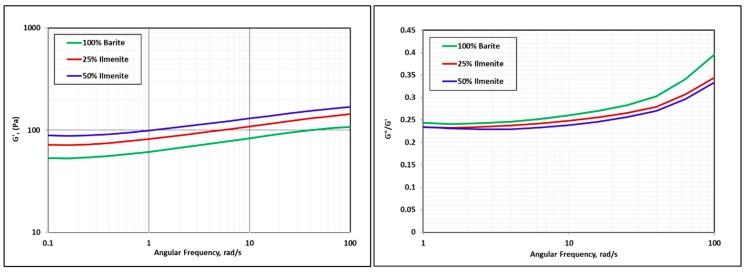
Effect of ilmenite on viscoelastic properties (250 °F).

Figure 10 compares the viscoelastic properties of the base fluid, 25 wt.% ilmenite, and 50 wt.% ilmenite measured at 250 °F. It was found that the storage modulus G′ was higher than the loss modulus G″ for all the drilling fluid samples, which means that the linear viscoelastic range was dominated by the elastic property. Adding ilmenite to the drilling fluid increased the storage modulus, reflecting higher elasticity and a more stable gel structure [39].

**Figure 11:**
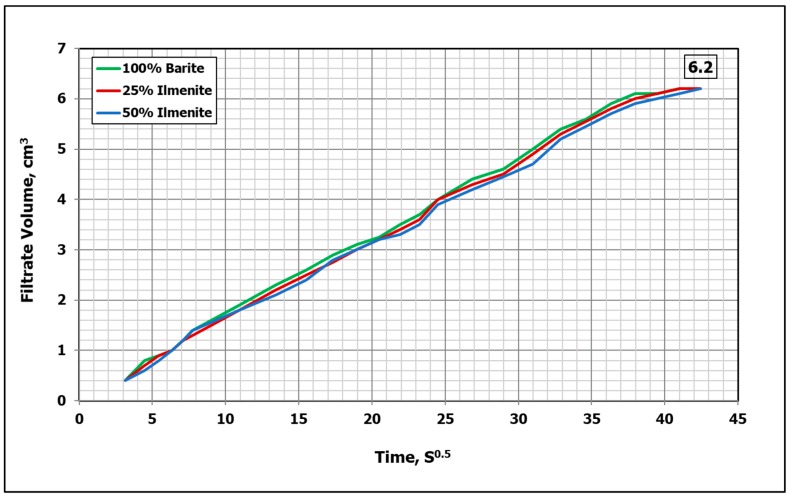
Effect of ilmenite on filtration performance of drilling fluid (250 °F).

**Figure 12:**
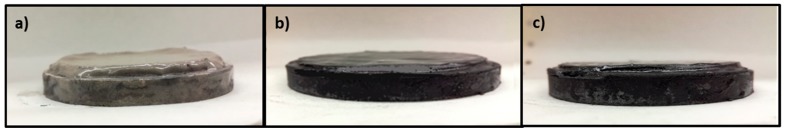
The resulting filter cakes: (**a**) 100 wt.% barite (2.59 mm), (**b**) 25wt.% ilmenite (2.56 mm), and (**c**) 50 wt.% ilmenite (2.51 mm).

An HPHT filtration test was conducted to evaluate the effect of combined weighting material on the filtration performance of the drilling fluid. Figure 11 compares the filtration performance of the base fluid, and the 25 wt.% and 50 wt.% ilmenite fluid samples at 250 °F and 300 psi differential pressure. It was found that adding up to 50 wt.% ilmenite (from the total weight of the weighting material) had no impact on the filtration performance. The total fluid filtrate was 6.2 cm^3^ and a 2.5-mm filter cake was formed in all fluid samples (Figure 12). Table 5 summarizes the filtration test results.

## 4. Conclusions

Extensive experimental work was conducted to evaluate the effect of combining barite–ilmenite weighting materials to mitigate the barite sag issue in water-based drilling fluid. From the results of this work, the following conclusions can be drawn:

- As the concentration of ilmenite was increased, the final density of the drilling fluid increased because of the high density of ilmenite, while the pH of the drilling fluid slightly decreased to reach 9.5 for 100 wt.% ilmenite. However, the pH value is still within the acceptable pH range (9–11) and it can be maintained by adding pH control additives.
- Adding ilmenite enhanced the stability of the drilling fluid and significantly reduced the sag factor for both static and dynamic conditions. The sag factor decreased as the concentration of ilmenite increased, and 50 wt.% ilmenite was adequate to prevent barite sag in both conditions. The sag factor dropped from 0.59 to 0.51 in static conditions, while VSST dropped from 1.75 to 0.33 in dynamic conditions.
- Ilmenite improved the rheology of the drilling fluid and maintained rheological properties under HPHT conditions. In contrast, a huge drop in rheological properties of the base fluid (without ilmenite) was observed because of the high temperature. Similarly, ilmenite enhanced the viscoelastic properties of the drilling fluid by increasing the elasticity of the drilling fluid, which validates the sag test results.
- From the HPHT filtration results, adding ilmenite to the base drilling fluid had no impact on the filtration performance and filter cake properties. The total filtrate volume was 6.2 cm^3^ for both fluids (100 wt.% barite and 50 wt.% ilmenite), while a 2.5-mm filter cake was formed in both cases.
- Based on the rheology and sag test results, 50 wt.% is the optimum concentration of ilmenite, and adding more than this concentration is not recommended as it would add more cost to the drilling operation.
- The combined barite–ilmenite weighting material could be a good option to safely kill or drill HPHT oil and gas wells with minimal sag tendency; however, more research work is needed to remove the composite filter cake in the case of drilling, because the removal process of the filter cake would be more complex as two different materials need to be dissolved. Therefore, a new approach to remove the composite filter cake is required.

## Figures and Tables

**Table 1:** Drilling fluid formulation used in this study.

Component	Amount (g)	Mixing Time (min)	Function
**Water**	245	-	Base fluid
**Defoamer**	0.08	1	Anti-foam agent
**Soda ash**	0.5	1	Maintain calcium concentration
**XC-polymer**	1.5	20	Viscosity control
**Bentonite**	4	10	Viscosity control
**Potassium hydroxide**	0.5	1	pH adjustment
**Starch**	6	10	Fluid loss control
**PAC-R**	1	10	Fluid loss control
**Potassium chloride**	20	10	Clay stabilization
**Calcium carbonate**	5	10	Bridging agent
**Barite**	350	10	Weighting material

**Table 2:** Properties of base drilling fluid.

Shear Rate (RPM)	Shear Stress (D.R.)	
600	95.8	
300	64	
200	52.5	
100	38.3	
6	17.9	
3	17.2	
**Density**	15	ppg
**PV**	31.8	cP
**YP**	32.2	lb/100 ft^2^
**Gel strength 10 s**	16	lb/100 ft^2^
**Gel strength 10/30 min**	28/28	lb/100 ft^2^

**Table 3:** Experimental conditions for sag test.

Static Sag Test		Dynamic Sag Test	
Temperature	250 °F	Temperature	120 °F
Pressure	500 psi	Pressure	Atmospheric pressure
Time	24 h	Time	30 min
Inclination	Vertical (45°)		

**Table 4:** Experimental conditions for the high-pressure/high-temperature (HPHT) filtration test.

Parameter	Description
Condition	Static
Differential pressure	300 psi
Temperature	250 °F
Time	30 min
Filtration medium	10-μm ceramic filter disc

**Table 5:** Summary for HPHT filtration test results.

Parameter	100% Barite	25% Ilmenite	50% Ilmenite
Filtrate volume (cm^3^)	6.2	6.2	6.2
Filter cake thickness (mm)	2.59	2.56	2.51
Filter cake weight (g)	23	21.4	19.92
